# Influence of different primary surgical techniques on long-term intraocular pressure and medication in glaucoma after congenital cataract surgery

**DOI:** 10.1371/journal.pone.0286318

**Published:** 2023-07-05

**Authors:** Alicja Strzalkowska, Piotr Strzalkowski, Julia V. Stingl, Norbert Pfeiffer, Alexander K. Schuster, Esther M. Hoffmann

**Affiliations:** 1 Childhood Glaucoma Center, Department of Ophthalmology, University Medical Center Mainz, Mainz, Germany; 2 Department of Ophthalmology, Helios HSK Wiesbaden, Wiesbaden, Germany; Alexandria University Faculty of Medicine, EGYPT

## Abstract

**Purpose:**

To assess long-time results of primary surgical treatment in children with glaucoma after congenital cataract surgery.

**Methods:**

A retrospective study of 37 eyes from 35 children with glaucoma after congenital cataract surgery, who underwent surgery between 2011 and 2021 at the Childhood Glaucoma Center, University Medical Center Mainz, Germany. Only children, who received a primary glaucoma surgery in our clinic within the given time (n = 25) and had at least one-year follow-up (n = 21), were included in the further analysis. The mean follow-up time was 40.4±35.1 months. The primary outcome was the mean reduction in IOP (in mmHg) from baseline to follow-up visits after the surgery, measured with Perkins tonometry.

**Results:**

8 patients (38%) were treated with probe trabeculotomy (probe TO), 6 (29%) with 360° catheter-assisted trabeculotomy (360° TO) and 7 (33%) with cyclodestructive procedures. IOP was significantly reduced after probe TO and 360° TO after 2 years, from 26.9 mmHg to 17.4 mmHg (p<0.01) and 25.2 mmHg to 14.1 mmHg (p<0.02), respectively. There was no significant IOP reduction after cyclodestructive procedures after 2 years. Both, probe TO and 360° TO led descriptively to eye drops reduction after 2 years, from 2.0 to 0.7 and 3.2 to 1.1. The reduction was not significant.

**Conclusions:**

In glaucoma after congenital cataract surgery, both trabeculotomy techniques, lead to good reduction of IOP after 2 years. There is a need for a prospective study with comparison to the use of glaucoma drainage implants.

## Introduction

The prevalence of congenital cataract is 4.24 per 10.000 people and is the leading preventable cause of childhood blindness [1].

Timely lentectomy in childhood [2] is crucial to prevent irreversible changes, such as nystagmus. Once this has manifested, normal visual development is no longer possible. 3–12 years after lens extraction, glaucoma after congenital cataract surgery can develop in 12–41% of cases [3–6], with earlier lentectomy associated with a higher risk [7]. Lifelong glaucoma screening is necessary as it can manifest later in life [5, 8–11]. Diagnosing glaucoma after congenital cataract surgery is particularly challenging. Measuring intraocular pressure (IOP) in young children is much more difficult due to reduced cooperation. Evaluation of the optic nerve head is demanding, because the pupil after lentectomy tends to stay small, despite the usage of dilating eye drops. Examinations usually used for adolescents and adults to diagnose glaucoma, such as visual field or optical coherence tomography (OCT) of the optic disc head, cannot be performed [12].

Treating glaucoma after congenital cataract surgery presents unique challenges [13]. Eye drop application does not allow long-term IOP reduction and is impractical in children. Some eye drops such as α2-adrenoceptor agonists are of concern in children because of potential side effects [14]. In most cases, long-term reduction of IOP is typically achieved through glaucoma surgery. 30% of patients require more than one glaucoma surgery. There is a wide range of possible glaucoma surgery techniques, but there is a lack of consensus regarding the optimal surgical procedure for glaucoma after congenital cataract surgery [15]. Among the most currently being used are: trabeculectomy and drainage implants, other possibilities are trabeculotomy, cyclodestructive surgery or goniotomy. Assessing the success of the operation is currently difficult, and thus makes it tough to evaluate the best surgical technique for aphakic children. As glaucoma after congenital cataract surgery is a rare disease, study collectives are small and have different follow-ups. Success rates for glaucoma after congenital cataract surgery vary based on the chosen surgical technique and success criteria, ranging from 0% to 40% for trabeculectomy [11, 15, 16] and up to 67% for Ahmed implant [15]. Long-time studies on surgical treatment in glaucoma after congenital cataract surgery in children surgical treatment is still insufficient.

Our clinical experience coincides with the experience of Bothun et al. that angle surgery, such as trabeculotomy is often effective, not only in primary congenital glaucoma [17] but also in glaucoma after congenital cataract surgery, when circular angle chamber synechia is absent [17, 18]. Trabeculotomy requires less time compared to drainage implantation, resulting in shorter anesthesia duration for children undergoing the procedure.

Our study aimed to present a safe alternative operation technique to drainage implantation or trabeculectomy for treating glaucoma after congenital cataract surgery in children.

## Materials and methods

A retrospective study of 37 eyes from 35 children with glaucoma after congenital cataract surgery, who underwent surgery between 2011 and 2021 at the Childhood Glaucoma Center, University Medical Center Mainz, Germany. Only children, who received a primary glaucoma operation in our clinic within the given time (n = 25) and had at least one-year follow-up (n = 21), were included in the further analysis. Surgery was indicated, if at least 2 criteria of The World Glaucoma Association Consensus were met: IOP above 21 mmHg, corneal findings such as Haab’s strie/megalocornea, optic disc excavation, increasing myopia or visual field loss (in older children). In cases where glaucoma after congenital cataract surgery was diagnosed late and the vision was severely impaired (worse than 1.9 on the logMAR scale), or when only one eye was affected in the advanced stage of the disease, or if the child had other health issues, we considered a cyclodestructive procedure as the initial treatment option. IRB (ethics committee Rheinland-Pfalz, Mainz, Germany) waived the need for IRB approval, because of the retrospective nature of the study. The study and data collection were in conformity with all country, federal, and state laws. The study was in adherence to the tenets of the Declaration of Helsinki. The mean follow-up time was 40.4±35.1 months.

The primary outcome was the mean reduction in IOP (in mmHg) from baseline to follow-up visits after the surgery, measured with Perkins tonometry. The following data were collected at the time of surgery and evaluated as well: demographics, number and type of surgical interventions, number of eye drops, cornea dimensions, corneal thickness and axial length. The surgical steps of trabeculotomies were described before [19]. Surgeries were performed by 3 experienced glaucoma specialists.

### Statistical methods

Categorical variables were presented as absolute and relative frequencies, whereas mean and standard deviation were computed for approximately normal-distributed continuous variables. Evaluation of data normality was performed using the Shapiro-Wilk test. Categorical variables were compared using Fisher’s exact test. Non-normally distributed continuous variables were compared using Mann-Whitney and Wilcoxon tests. For multiple comparisons, non-parametric Kruskal-Wallis test and post hoc Dunn’s test were used. All statistical tests were two-sided and *p*-value <0.05 was considered statistically significant. Statistical analysis was performed using GraphPad Prism9 (GraphPad Software, San Diego, USA) for Mac.

## Results

### Characteristics

21 eyes with glaucoma after congenital cataract surgery without previous glaucoma surgery and at least one-year follow-up were included in this study. Table 1 shows the characteristics of the study population.

**Table 1:** Demographics of study population.

Age at presentation (years)	
Mean±SD	6.0±4.05
Range	0 (3 months)-13
Preoperative IOP (mmHg), Perkins	
Mean±SD	25.6±8.85
Range	11–43
Number of preoperative eye drops	
Mean±SD	2.41±1.46
Range	0–4
Corneal vertical diameter (mm)	
Mean±SD	11±0.96
Range	9.0–12.0
Corneal horizontal diameter (mm)	
Mean±SD	11.4±0.87
Range	10.0–12.5
Corneal thickness (mm)	
Mean±SD	698±80.1
Range	533–850
Axial length (mm)	
Mean±SD	22.2±1.33
Range	19.9–24.4

### Frequency of operation technique

Out of 21 patients in our study, 8 patients (38%) were treated with probe trabeculotomy (probe TO), 6 patients (29%) underwent 360° catheter-assisted trabeculotomy (360° TO) and 7 patients (33%) received cyclodestructive procedures. There was no statistical difference between those three groups concerning age, gender, preoperative IOP and number of preoperative eye drops, see Table 2.

**Table 2:** Demographics of study population, divided into three groups based in the surgical technique.

	Probe TO	360° TO	Cycloclodestructive procedure	p value
Age at presentation (years)				
Mean±SD	6.5±3.6	6.0±4.7	5.4±4.6	0.88
Gender				
Female (%)	4 (50%)	4 (66.7%)	3 (42.9%)	0.68
Preoperative IOP (mmHg)				
Mean±SD	26.9±9.2	25.2±6.9	24.0±11.8	0.86
Number of preoperative eye drops				
Mean±SD	2.0±1.9	3.2±0.4	2.0±1.4	0.37

### Intraocular pressure

Mean preoperative IOP measured with Perkins was 25.6 mmHg for the entire study population. At the last follow-up, a significant reduction in IOP was observed, with the mean IOP decreasing to 16.9 mmHg (-8.7 mmHg).

After probe TO, there was a significant reduction in IOP from 26.9 mmHg to 16.4 mmHg after 1 year (-10.5 mmHg, p = 0.01) and to 17.4 mmHg after 2 years (-9.5 mmHg, p = 0.02), resulting in a 39.0% reduction in IOP after 1 year and 35.3% reduction after 2 years.

Following 360° TO, the IOP decreased from 25.2 mmHg to 14.8 mmHg after 1 year (-10.4 mmHg, p = 0.02) and 14.1 mmHg after 2 years (-11.1 mmHg, p = 0.04), corresponding to a 41.3% reduction in IOP after 1 year and 44% reduction after 2 years.

**Fig 1:**
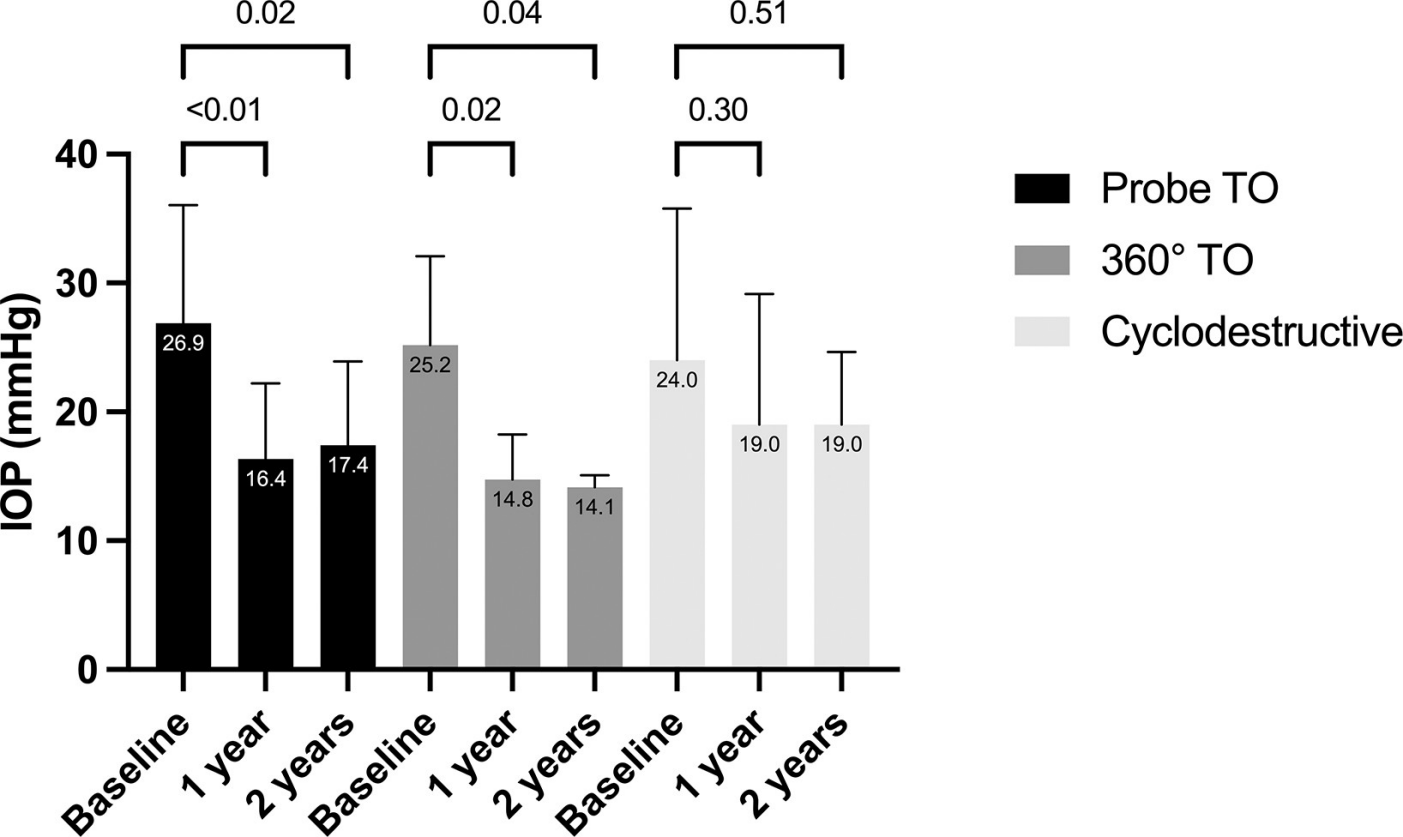
IOP reduction after probe TO, 360° TO, cyclodestructive procedure in glaucoma after congenital cataract surgery.

Cyclodestructive interventions lowered the IOP from 24.0 mmHg to 19.0 mmHg after 1 year (-5.0 mmHg, p = 0.30) and 19.0 mmHg after 2 years (-5.0 mmHg, p = 0.51), leading to a 20.8% reduction in IOP after both 1 year and 2 years. However, the reduction was not statistically significant. The IOP reduction of these three surgical techniques is illustrated in the Fig 1.

There were no statistically significant differences observed in IOP reduction at both 1 and 2 years when comparing the probe TO, 360° TO and cyclodestructive procedures.

### Reduction of glaucoma medication

The preoperative number of glaucoma medication was recorded as 2.4±1.5 for the entire study population. At the last follow-up, a significant reduction was observed, with the average number of medications decreasing to 0.92.

After probe TO, the number of eye drops decreased from 2.0 to 0.7 after 1 year and to 0.9 after 2 years. Similary, after 360° TO, there was a reduction of eye drops from 3.2 to 1.2 after 1 year and 1.0 after 2 years.

Cyclodestructive interventions led to a decrease in the number of glaucoma medication from 2.0 to 1.0 after 1 year and 1.0 after 2 years.

**Fig 2:**
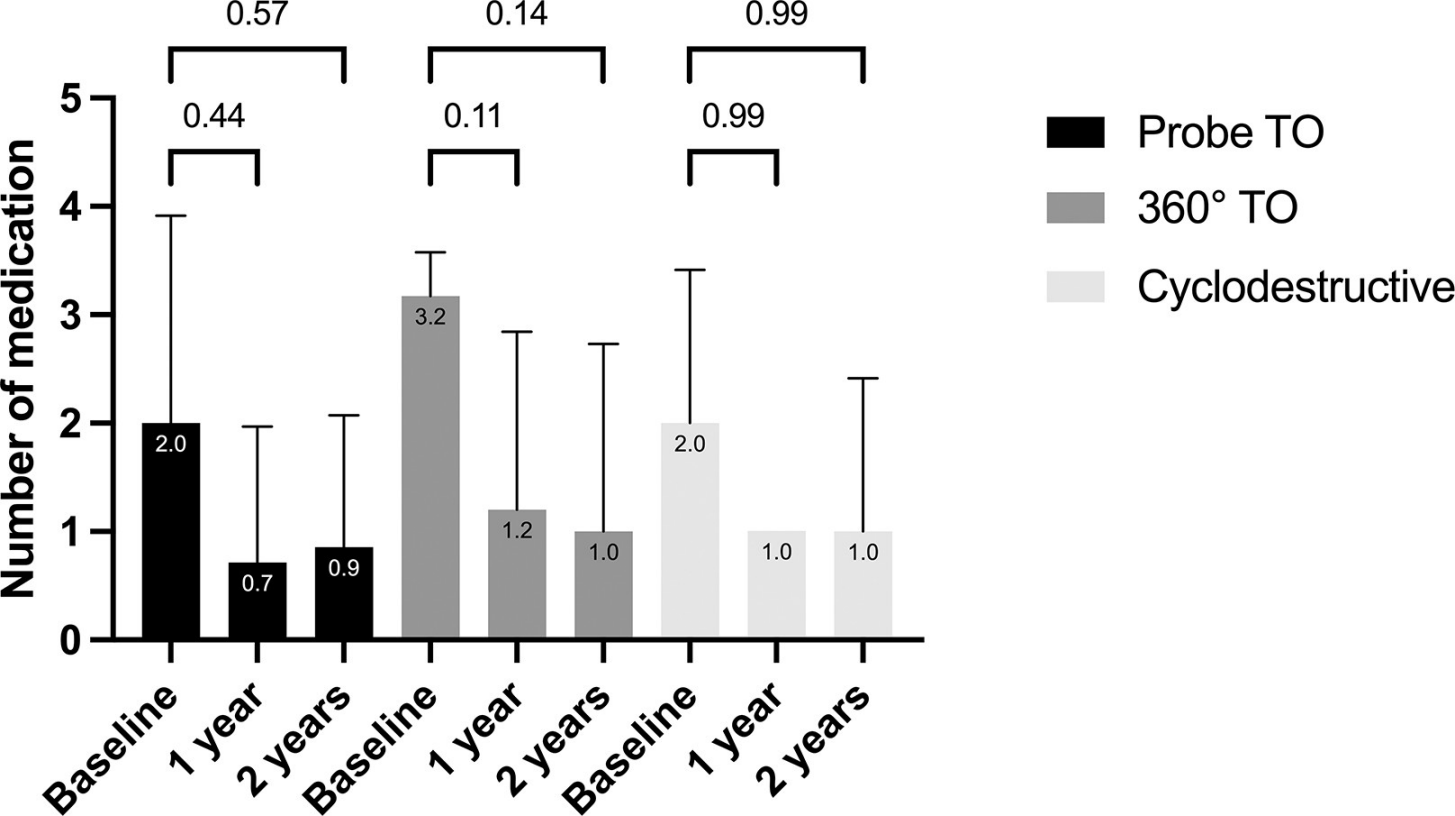
Number of glaucoma medication after probe TO, 360° TO, cyclodestructive procedure in glaucoma after congenital cataract surgery.

After dividing the entire study population into three groups based on the surgical technique, the reduction in the number of glaucoma medication was no longer statistically significant. The glaucoma eye drop reduction after those three operations techniques are shown in the Fig 2.

4 Patients after probe TO did not need any eye drops to control glaucoma, in comparison to 2 patients after 360° TO and 1 patient after cyclodestructive intervention.

### Reoperations

Reoperation was conducted, when IOP was above 21 mmHg and there was a sign of progression in: corneal findings, optic disc excavation, myopia or visual field 4–6 weeks after the first operation, although the maximal possible local IOP lowering medication was given.

During the 3-year follow-up period, two children having had a cyclodestructive procedure and one child after probe TO required reoperation. One patient who underwent cyclodestructive procedure needed another cyclodestructive procedure within the first year, subsequently an Ahmed drainage implant was inserted to control the IOP. The second patient received a Baerveldt drainage implant within the first two years, which led to IOP control for the follow-up period. The child having had probe TO received 360° TO within the first three years, which resulted in good pressure control thereafter.

## Discussion

This study examined the outcomes of probe TO, 360° TO and cyclodestructive interventions for managing glaucoma after congenital cataract surgery. Significant reduction in IOP were observed after probe TO and 360° TO, but not after cyclodestructive procedures.

Based on a recent review, the most appropriate surgical technique in glaucoma after congenital cataract surgery remains uncertain [20].

According to the literature, drainage implants and trabeculectomy are commonly performed surgeries to reduce IOP in glaucoma after congenital cataract surgery. However, these techniques have their limitations. Drainage implants can be associated with sight-threatening complications [21], while trabeculectomy alone may not achieve IOP<21 mmHg without glaucoma eye drops and clinically stable glaucoma [16]. To improve success rates, the addition of the mitomycin C after trabeculectomy has been attempted, but it comes with increased risk of complications [22]. Cyclodestructive procedures, on the other hand, provide only short-term IOP reduction [23].

Promisingly, minimally invasive glaucoma surgeries (MIGS) are expected to play a role in glaucoma management after congenital cataract surgery, following encouraging results in a few studies [24, 25].

In our study, significant reduction in IOP was observed after probe TO and 360° TO. The IOP decreased from 26.9 to 16.4 mmHg and from 25.2 to 14.8 mmHg after one year, respectively. These findings were consistent with a prospective study by Pakravan et al., which compared IOP reduction after trabeculectomy and Ahmed implant in children with glaucoma after congenital cataract surgery. In their study, the preintervention IOP was 31.0 mmHg and decreased to 14.7 after trabeculectomy and 14.4 mmHg after Ahmed implant, with a follow-up duration of 13.1 to 14.8 months [15]. Kirwan et al. reported an IOP reduction after Ahmed implant in 19 eyes from 13 children, from a baseline of 36 mmHg to 18 mmHg, over an average follow-up of 32 months. It’s worth noting that nine eyes in their study had previous glaucoma surgeries, unlike the eyes in our study, which only underwent lentectomy [13]. Mandal et al. reported slightly lower IOP reduction after trabeculectomy, with a preoperative IOP of 34.2 mmHg reducing to 18.4 mmHg in a group of 23 eyes from 19 children, with a mean follow-up of 24.2±17.9 months. 9 children received MMC intraoperatively. The patient population in their study was relatively older compared to ours, with an average age of 9.6±6.5 years versus 6.0±4.05 years [11]. In the study by Beck et al., using 360° suture TO, the IOP reduced from 33.8±5.0 to 22.9 ±6.7 mmHg at one year [26].

Three independent studies evaluating the IOP reduction following drainage implantation consistently demonstrated similar results. In the first study, the IOP decreased from a baseline of 36 mmHg to 15 mmHg [27]. The second study reported a reduction from 32.66±6.73 mmHg to 16.54 ± 2.75 mmHg (p < 0.001) [28]. Similarly, the third study conducted by Geyer observed a decrease from 35.8±7.4 mmHg to 18.7±6.5 mm Hg at the last visit [29]. Notably, the preoperative IOP in all these studies was higher compared to our study. However, the postoperative IOP achieved similar levels when compared to our findings.

El Sayed et al. conducted a prospective study evaluating the outcomes of two-site trabeculotomy. Their findings showed a significant reduction in IOP from 22.3 to 14.1 mmHg one year after the surgery [30]. Similarly, Bothun et al. conducted a retrospective study including 14 eyes, where different surgical approaches were employed (goniotomy, goniotomy followed by trabeculotomy, and trabeculotomy alone). The study population demonstrated a reduction in mean IOP from 25.0±10.0 at baseline to 22.0±4.0 mmHg over a follow-up period of 55 months [18]. These results align with our study, where the mean preoperative IOP measured with Perkins was 25.6 mmHg for the entire study population. At the last follow-up, a significant reduction in IOP to 16.9 mmHg was observed.

In our study, although the use of antiglaucoma eye drops was still necessary one year after probe TO and 360° TO, a notable reduction in the number of topical medications was observed. After probe TO, there was a reduction in eye drops from 2.0 to 0.7 and after 360° TO the reduction was from 3.2 to 1.2 after one year. This reduction was not statistically significant. In the study by Pakravan et al., it was reported that the number of antiglaucoma medications decreased from 3 to 1.67 after trabeculectomy and 3.3±0.5 to 1.6±0.51 after Ahmed implant [15]. Similarly, in El Sayed’s study, a significant reduction in the number of glaucoma medications was observed from 2.34 to 0.9 after one following two-site trabeculotomy [30]. After the drainage implantation, approximately 80% of patients still required glaucoma medication [29].

In 3 years, 2 children who had cyclodestructive procedures and 1 child who had probe TO needed reoperation. However, in 18 children, the IOP was effectively controlled throughout the follow-up period. In the study by Kiwan et al., 3 out of 19 eyes maintained good IOP control for over 6 years [13]. In the retrospective study by Asrani et al., 78.6% (11 out of 14) of patients achieved successful IOP control with a single operation, without the need of glaucoma medications [5]. Another study involving drainage implantation reported a reoperation rate of 22% within a 5.5-year follow-up period [27].

Our study has several limitations, primary due to its retrospective design. The selection of the surgical procedure was based on the surgeon’s preference, with trabeculotomy being the preferred method due to the extensive experience in our glaucoma center. It should be noted that glaucoma drainage implantation is not a primary procedure for managing glaucoma after congenital cataract surgery at our clinic. Nonetheless, this study provides valuable insights for making informed decisions in the clinic setting regarding the appropriate surgical treatment for glaucoma after congenital cataract surgery. Furthermore, it contributes to the establishment of treatment recommendation for this rare yet significant condition.

## Conclusion

Both trabeculotomy techniques have demonstrated significant long-term reduction in intraocular pressure (IOP) after 2 years in patients with glaucoma after congenital cataract surgery. These surgical methods offer a safe alternative with low reoperations rates, making them viable first-line surgical therapies. However, there is a need for a prospective study that compress these trabeculotomy techniques to drainage implantation to further evaluate their effectiveness and determine the optimal treatment approach for this patient population.

## Supporting information

S1 Table (XLSX)
